# Oral anticoagulation is preferable to injected, but only if it is safe and effective: An interview study of patient and carer experience of oral and injected anticoagulant therapy for cancer-associated thrombosis in the select-d trial

**DOI:** 10.1177/0269216318815377

**Published:** 2018-11-29

**Authors:** Ann Hutchinson, Sophie Rees, Annie Young, Anthony Maraveyas, Kathryn Date, Miriam J Johnson

**Affiliations:** 1 University of Hull, Hull, UK; 2 University of Warwick, Coventry, UK

**Keywords:** Thrombosis, anticoagulants, neoplasms, injections, tablets, interview, direct oral anticoagulants, low-molecular-weight heparins

## Abstract

**Background:**

Cancer patients have a four- to fivefold greater risk of thrombosis than the general population. Recommended treatment for cancer-associated thrombosis is 3–6 months of low-molecular-weight heparin. The ‘select-d’ trial is an open-label, randomised, multi-centre pilot trial in patients with cancer-associated thrombosis, utilising dalteparin (low-molecular-weight heparin) versus rivaroxaban (a direct oral anticoagulant), to assess effectiveness and safety.

**Aim:**

To explore patient and informal carers’ experiences of cancer-associated thrombosis and their experience and understanding of the risk–benefit of thrombosis treatment.

**Design:**

Qualitative substudy of the select-d trial, using semi-structured interviews. Interviews were audio-recorded and transcribed. Data were analysed using Framework Analysis.

**Participants:**

Participants were purposively sampled (*n* = 37 patients; 46% male; age 40–89; 9 with carer present).

**Results:**

Three themes were found: experience of cancer-associated thrombosis, experience of anticoagulation and risk–benefit balance of the two modes of administration. Some were shocked by their thrombosis diagnosis (most were unaware of their risk), but others found it insignificant compared with cancer. Most patients found tablets more convenient, but injections were acceptable in the context of having cancer. While most were happy to follow medical advice, others weighed preference on the basis of effectiveness.

**Conclusion:**

Lack of awareness of thrombosis risk is concerning; cancer patients must be informed to enable prompt help-seeking. Tablets could provide a welcome choice for patients if there is equivalent risk–benefit to injected anticoagulants. Patients trust their clinicians to tailor their treatment. Future research could explore the effect of routine information giving about the risk of thrombosis.

**What is already known about the topic?**

- Cancer patients are at an increased risk of thrombosis.
- Current guidance for treatment is injected anticoagulants although there is some doubt as to the long-term acceptability of injections to patients.
- Many cancer patients are unaware of their increased risk of thrombosis or of the symptoms that should prompt seeking medical attention.

**What this paper adds?**

- Cancer patients find injected anticoagulants acceptable in the context of cancer, especially when given support to overcome initial anxieties.
- Patients find taking tablets easier, but would only choose tablets over injections if found to be as safe and effective as injected anticoagulants.

**Implications for practice, theory or policy**

- Cancer patients must be informed of their increased risk of thrombosis and the symptoms for which they should seek help.
- Rivaroxaban tablets could be offered as a choice when there are sufficient robust data to support the risk–benefit balance.

## Introduction

Cancer-associated thrombosis is the second highest preventable cause of cancer mortality, with the greatest risk of death in the first 3 months of diagnosis.^1^ Distressing complications, such as venous thromboembolism recurrence and bleeding, are also more common among cancer patients.^1–3^ It is associated with significant symptoms and clinicians find it challenging to diagnose and treat, particularly in advanced disease.^4–6^

Clinical guidelines for cancer-associated thrombosis recommend 3–6 months of daily subcutaneous injection of low-molecular-weight heparin;^7–9^ there is a 50% relative risk reduction in recurrent venous thromboembolism with low-molecular-weight heparin compared to vitamin K antagonists, without an increase in bleeding.^10–13^ Direct oral anticoagulants are currently recommended in guidelines for the cancer population only if low-molecular-weight heparin is not tolerated.^7,14^

Despite high-quality evidence supporting cancer-associated thrombosis treatment, there are few data about patient experience. A systematic review and synthesis^15^ found only five studies.^16–20^ These showed poor knowledge of the risk and symptoms of cancer-associated thrombosis, but challenges in relation to thrombosis and its treatment added significantly to the life disruption caused by the cancer. Despite concerns that daily injections were too invasive, these are viewed by patients as an acceptable trade-off against a serious, life-threatening condition.^18,21^

A choice-based conjoint experiment study assessed preferences for anticoagulation in people with cancer-associated thrombosis with no previous experience of direct oral anticoagulants.^21^ Participants valued safety and effectiveness over route of administration, ranking ‘minimal interference with cancer treatment’ and ‘low rates of recurrence and risk of major bleeds’ as most important.

The select-d trial (Anticoagulation Therapy in SELECTeD Cancer Patients at Risk of Recurrence of Venous Thromboembolism (ISRCTN: 86712308)) is a 6-month randomised, multi-centre pilot study comparing dalteparin (a low-molecular-weight heparin) with rivaroxaban (a direct oral anticoagulant). After 6 months, eligible participants were re-randomised to either rivaroxaban or a placebo tablet for a further 6 months.

This substudy aimed to explore patient and carer experience of cancer-associated thrombosis and of different modes of anticoagulant administration.

## Methods

### Methodology

The aim of this study was to inform clinical practice. Framework Analysis^22^ was therefore chosen as it adopts the ontological position of ‘subtle realism’^23^ in which the social world is seen as existing independently of subjective understanding, albeit accessible through the participants’ interpretations. Well suited to analyses of qualitative data in a multidisciplinary team, and when the aim is to produce accessible evidence on which to base clinical recommendations, it allows a balance between summarising and reducing the data, on the one hand, and retaining participants’ own words and meanings on the other.^24^

### Design

#### Ethical approval

Ethical approval, including for the method of consent, was gained from Coventry and Warwickshire Research Ethics Committee in October 2015 (REC No. 13/WM/0017).

#### Sampling of participants

A purposive sample from different groups of select-d trial patient-participants, with at least 2 months of treatment, was identified (Table 1). Group 3 was particularly important as they had experience of both injectable *and* oral anticoagulants.

**Table 1.** Sampling framework for select-d qualitative substudy.

Group	Experience
1	Dalteparin (injections) only
2	Rivaroxaban (tablets) only
3	Dalteparin followed by rivaroxaban
4	Stopped early while receiving dalteparin (injections)
5	Stopped early while receiving rivaroxaban (tablets)

#### Recruitment

Potential patient-participants from a variety of sites were identified from the trial database by the interviewers (A.H. and S.R.) and then approached by the relevant local site research nurse to gain consent to participate in the substudy. Subsequently, the interviewers contacted the patient-participants to arrange a suitable time for the interview. The family carers of participants, if one was identified, were also invited to interview with the patient-participant. None refused to participate or dropped out.

#### Data collection

Semi-structured interviews (lasting 8–62 min) were conducted by S.R. and A.H. by telephone or in person in the patient’s home following a topic guide derived from the literature and team expertise (see Appendix 1). Demographic data were only recorded for patients. Interviews were audio-recorded and transcribed verbatim.

#### Reporting

This article was reported in accordance with the Consolidated criteria for reporting qualitative research (COREQ) checklist.^25^

### Analysis

The defining characteristic of the Framework method is the matrix output which displays cases in rows and codes in columns, with cells containing data excerpts or summarised data. Themes and patterns are identified and explored both across and within cases, and relationships and connection between categories can be investigated. A systematic procedure was followed.^24^ Data were line-by-line coded and codes were grouped into categories – clusters around a particular concept.^24^ Computer-Assisted Qualitative Data Analysis Software was used to aid the data analysis, carried out by both A.H. and S.R., with input from M.J., A.M., and A.Y. Iterations of the framework were developed as the coding progressed, through discussions among the researchers.

## Results

In total, 37 interviews were conducted between October 2015 and September 2016 (age range 60–79; 46% men; 100% White British; Table 2). The patient-participant group had various primary cancer sites including breast, lung and colorectal cancer, and both deep vein thrombosis and pulmonary embolism. Approximately half the patients had metastatic cancer, one had haematological malignancy and the remainder had early or locally advanced cancer. Nine carer-participants were interviewed with a patient-participant.

**Table 2.** Sample characteristics.

Characteristic	Number (%)
Group	
1 (injections)	10 (27)
2 (tablets)	11 (30)
3 (both)	9 (24)
4 (injections stopped early)	4 (11)
5 (tablets stopped early)	3 (8)
Age	
40–49	2 (5)
50–59	2 (5)
60–69	18 (49)
70–79	14 (38)
80–89	1 (3)
Gender of patient	
Female	20 (54)
Male	17 (46)

### Experience of cancer-associated thrombosis

#### Reaction to diagnosis

Only 7/37 patient-participants had been aware of their increased cancer-associated thrombosis risk, either informed by their healthcare team or due to previous experience of venous thromboembolism in family members. Most patient-participants were unaware and had attributed their symptoms to side effects of cancer or its treatment which delayed presentation, diagnosis and treatment for the cancer-associated thrombosis:

I’d been in pain with my leg for a good week or so but you just think it’s part of the cancer. P2 (Group 1)

I was in sort of blissful ignorance, I was thinking ‘Oh maybe it’s part and parcel of this cancer, maybe it’s the lymph nodes causing the breathlessness’. P25 (Group 2)

Some participants, patients and carers, were distressed when they realised it was life-threatening, confronted with the possibility of a sudden death from something other than cancer:

I was utterly astounded, quite honestly … I thought ‘Wow, my God, it could have killed me’. P3 (Group 2)

Some described it as ‘a bit of a blow’ (P22) on top of the cancer and found it very worrying. However, not all patients reacted this way, cancer-associated thrombosis viewed as ‘part and parcel’ (P23) of having cancer and described feeling ‘laid back’ (P15) or even ‘sanguine’ (P2) about the venous thromboembolism:

The fact I’d got a few tiny blood clots on my lungs was a minor detail [laughter] to be honest. P25 (Group 2)

Quite honestly, it was a non-event. Having been through prostate … After that really, on a scale of one to ten, it was about a two. It was shrugged off, virtually. P24 (Group 1)

I don’t think I had a lot of time to think about it because I was far more, I was concentrating far more, or feeling more affected by the chemotherapy. P11 (Group 1)

These views were expressed by both those with and those without symptomatic venous thromboembolism, and by those with pulmonary embolism or a deep vein thrombosis.

#### Impact of cancer-associated thrombosis on everyday life

Few patient-participants talked about the impact of cancer-associated thrombosis on everyday life. Those with asymptomatic incidental pulmonary embolism reported no impact on daily living. Some patient-participants with symptomatic cancer-associated thrombosis described impact from initial symptoms but resolving quickly with anticoagulation. They trusted in their medical care and felt safe that the cancer-associated thrombosis was being treated:

At least it had been found and it was something that could be treated. P20 (Group 2)

Some were worried about cancer-associated thrombosis recurrence, whereas others, although aware of the possibility of recurrence, took a pragmatic stance saving energy to deal with cancer treatment. Recurrence would be faced if necessary knowing the warning symptoms to trigger early help-seeking. Again, trust in their healthcare team eased their worries.

#### Experiences of anticoagulation therapy among cancer-associated thrombosis patients and their carers

##### Experience of taking direct oral anticoagulants

The experience of taking direct oral anticoagulants was set within the context of the many medications needed as part of cancer treatment. Tablets were seen generally as being straightforward to take. As most patients had an established routine for their cancer medication, the direct oral anticoagulant fitted in relatively easily:

I felt quite relieved that there was something that was happening every day to stop it happening again. C11 (Group 1)

However, some found the need to take it with food and at the same time every day difficult.

##### Experience of having low-molecular-weight heparin

The experience of having low-molecular-weight heparin was also set within the context of cancer treatment, which often involved injections and other invasive treatments. There were unwanted effects with injected low-molecular-weight heparin, but these were acceptable, and most found it straightforward to self-inject appreciating that they were contributing to their treatment by doing the injection. Like the direct oral anticoagulant group, patients developed a routine:

It was just another injection that was happening. P4 (Group 1)

You’re just feeling that you’re actually doing something, it’s a step forward. It’s contributing, me stomach’s not good at this moment, but if I keep injecting me leg’s going down, I can see, look, it’s turning back to what it were. P4 (Group 1)

Although some patient-participants had initial worries about their ability to do the injections, they adjusted quickly and only one patient-participant reported being unable to self-inject. In this case, a community nurse was able to support the patient which was acceptable despite causing inconvenience.

Carer-participants had mixed responses to being asked to help with injections. Some felt unable to inject their loved one because they felt too squeamish or did not want to hurt the patient, and one felt it would be too big a commitment. However, several were happy to contribute and helping in a difficult situation:

It’s difficult; I don’t know what to say sometimes. I suppose when I’m giving the injections perhaps it was a way out for me to think yeah, well at least I’m trying to do something. C35 (Group 3)

In these cases, the carers found the support from hospital staff invaluable, and they developed a routine with the patient.

We’re always early in the morning so I make the tea, I take a cup of tea up to (Patient name) she has a bit of tea, relax, and then I inject her. C35 (Group 3)

Patient-participants with experience of both injections and tablets varied in their views of the relative merit of the two methods of anticoagulation. Some were positive about the tablets compared to their previous experience of injections, while others were ambivalent and prepared to accept the injections despite the drawbacks.

I suppose taking a tablet is less problematic. But again, the injections didn’t worry me at all. P32 (Group 3)

I prefer the tablets, but the injections didn’t bother me. P18 (Group 3)

One patient could not swallow tablets and so preferred injections.

#### Approaching the risk–benefit balance of two different ways of anticoagulant administration

Many patients and carers seemed unaware of the relative effectiveness of the two treatments and were happy to simply comply with their doctors’ instructions:

I just do as I was bid. I have no medical knowledge at all and I just do as they say. P28 (Group 1)

Others weighed preference on the basis of effectiveness and adverse effects such as bruising; these participants had an appreciation that rivaroxaban was still being tested. The judgements were that a tablet would be easier and preferable but only if equally effective and safe, if not, the inconvenience associated with the injections was counteracted by the known effectiveness of the injections:

If a tablet would serve the same purpose then I would certainly sooner take a tablet, but … if the injections are an advantage then it’s worth putting up with the discomfort. P11 (Group 1)

If given the choice of equality of effect I would be preferring pills … If I was told that the pills would be less effective than the injections would I inject myself? And the answer is probably ‘Yes’. P31 (Group 3)

Overall, the feeling was that if the tablets and injections were equally effective, then the tablets were more straightforward and therefore preferable. However, if the injections were shown to be more effective, then the drawbacks such as bruising were an acceptable trade-off and injections would be preferable to the tablets.

## Discussion

### Main findings of the study

Most patients were unaware of their increased risk of cancer-associated thrombosis and of the symptoms, resulting in misattribution of symptoms and delayed help-seeking. Some patients were shocked or worried by their cancer-associated thrombosis; however, many were philosophical in the context of cancer. The reported impact of cancer-associated thrombosis was of a short-term nature, quickly resolving with treatment.

In the context of cancer, daily injections despite some drawbacks were not viewed as onerous. Many were happy to simply follow medical advice, but some took relative effectiveness into account. Tablets were considered to be easier to take, but injections were acceptable if they were more effective.

### What this study adds

The lack of awareness of the increased risk and symptoms of cancer-associated thrombosis is consistent with previous research^15,17,19^ and the All-Party Parliamentary Thrombosis Group findings in the United Kingdom.^26^ Misattribution of symptoms was also found by others.^19^ Patients are not the only group to be suboptimally aware. A survey of oncologists found a quarter of respon-dents did not recognise the risk of cancer-associated thrombosis.^27^ This study emphasises further the need for raised awareness of the risk and symptoms of cancer-associated thrombosis among clinicians and patients.^28^ Our data support recommendations^26,28,29^ that all people with cancer be made aware of cancer-associated thrombosis risk and venous thromboembolism symptoms, to aid timely help-seeking.

Most patients in this sample took the experience of cancer-associated thrombosis in their stride, although a few were shocked by it being consistent with some previous findings;^17^ two previous studies found that many patients responded negatively to their cancer-associated thrombosis diagnosis.^18,19^ This difference may be because we recruited from a selected population already prepared to take part in a trial.

Research regarding thrombosis in patients without cancer found the experience to be traumatic for many.^30–32^ Participants were young and considered themselves healthy before their diagnosis. Our participants found cancer-associated thrombosis to be less disruptive. This can be understood with reference to the theory of ‘biographical disruption’ wherein illness produces a ‘fundamental re-thinking of the person’s biography and self-concept’^33^ (p. 169). For our sample, it may have been experienced as a ‘continuity’ of biography, rather than a disruption.^34^ In other words, being diagnosed with a very serious illness and already having made the transition into patient-hood was perhaps protective against the impact of venous thromboembolism on identity and everyday life.

Patients in our study found that injected low-molecular-weight heparin was acceptable, as has been reported previously.^16,18^ Our data showed that injected anticoagulation therapy should be understood in the context of cancer. Although self-injecting and developing a routine (which patients did for tablets too) can be seen as forms of ‘illness work’,^35^ this work was perceived as minor in the context of the significant burden of work involved in being a ‘cancer patient’.

Most patient-participants trusted in the care of their clinicians and followed their advice over choice of medication without taking into account the relative effectiveness themselves. Therefore, the onus is on clinicians to know the guidelines and to prescribe accordingly.^28^ Presently, guidelines endorsed by the International Society on Thrombosis and Haemostasis^8^ recommend the use of low-molecular-weight heparin, but noting that some patients may find injections burdensome. Some patients and carers have initial worries over administering injections, but these can be assuaged with guidance on how to do injections and information on likely discomforts and how to deal with them. Others did not wish to self-inject but a carer may be prepared to, or a community nurse can administer the injections.

The preference for tablets shown by many of our participants is consistent with previous research,^18,21^ but this preference is not without qualification. Preference for oral administration is rated lower than the other factors, for example, effectiveness and safety.^21^ Therefore, while patients do generally prefer tablets, the route of administration is only one consideration. Patients accepted injections, if they were recommended by their clinician, or they understood them to be more effective and safe.

We support the recommendations of the All-Party Parliamentary Thrombosis Group^26^ that all National Health Service (NHS) trusts develop a cancer-associated thrombosis treatment policy based on the latest guidelines,^8^ taking into account the qualitative research demonstrating the patient-acceptability of low-molecular-weight heparin.^16,18^

### Future research

Future research could explore the effect of increasing cancer patients’ awareness of cancer-associated thrombosis on help-seeking behaviour and experience. The effects of giving information to patients and carers on how to do injections and handle the likely drawbacks on the acceptability of low-molecular-weight heparin could also be evaluated. Finally, contemporaneous data on oncologists’ awareness of cancer-associated thrombosis and anticoagulation prescribing practice would be useful.

There are two key issues. First, are direct oral anticoagulants as effective in cancer-associated thrombosis as low-molecular-weight heparin? The preliminary findings of the select-d randomised trial have been presented at the 59th American Society for Haematology meeting in Atlanta,^36^ and another trial reported in full.^37^ Data are promising in terms of effectiveness, although a careful comparison of the study populations is needed with regard to stage of cancer: those with more advanced or metastatic disease have a higher incidence of recurrence.^38^ Most select-d participants had more advanced or metastatic disease and recurrence was the same as low-molecular-weight heparin. However, in Raskob et al.,^37^ recurrence rate was reduced in the direct oral anticoagulant arm, but only half had metastatic disease and only third had recurrent cancer.

Second, are direct oral anticoagulants as safe as low-molecular-weight heparin? The emerging data show increased total bleeding, and in Raskob et al.,^37^ the reduced recurrence rate appeared to be at the expense of increased major bleeding (6.9% vs 4.0%). Although patient-participants in this substudy expressed preference for equal effectiveness and reported low-molecular-weight-heparin-related adverse effects, they mentioned little about bleeding. This is in contrast to existing literature where bleeding is described as a terrifying event. However, none of these patient-participants had experienced a major bleed, and only a few had experienced minor bleeds; therefore, it is unsurprising that few raised concerns about bleeding. They did, however, talk a lot about other drawbacks of low-molecular-weight heparin administration in the context of these being an acceptable payoff for an effective treatment. We do not know what information they had been given about risk of bleeding and they had little knowledge about the risk of cancer-associated thrombosis. We therefore cannot conclude that patient-participants were not worried about bleeding risk.

### Strengths and limitations of the study

This study included patient-participants with a variety of cancer primary sites, treated in many NHS sites. It is the first study to interview patients *and* carers with experience of taking direct oral anticoagulants.

Many interviews were conducted by phone with the inherent inability to register non-verbal cues such as body language. However, ‘non-visual paralinguistic cues’ can be just as useful as facial expressions and body language.^39^ The method of interview does not appear to have influenced the content or quality of the data. Patients and carers may have responded differently if interviewed separately.^40^ Conversely, interviewees may feel safe together and facilitate rapport-building.^41^ The views of the carers could be included, and patients and carers could reflect together on their experiences.

Patient-participants had already consented to take part in a clinical trial, and so this represents a pre-selected group who may not be fully representative. It was difficult to recruit patients who had stopped their medication early, and so their voices may not be fairly represented. All participants identified as White British, and results may have included different opinions had we a more ethnically diverse sample.

The two interviewers (A.H. and S.R.) were non-clinicians with no investment or clinical opinion on either drug. Nevertheless, the other researchers are clinicians with biases from clinical experience, possibly seen in the interpretation of the data. The chief investigator of the select-d trial (A.Y.) was on the qualitative substudy team. However, the only members of the team immersed in the data collection and analysis were A.H. and S.R. This study was funded by Bayer; however, they had no input into the analysis and interpretation of the data.

## Conclusion

Patients’ lack of awareness of cancer-associated thrombosis is concerning. Cancer patients should be informed of this risk and related symptoms to enable prompt help-seeking. Most patients found tablets more convenient, but low-molecular-weight heparin was acceptable in the context of cancer and its treatment despite drawbacks. Oral anticoagulants could provide a welcome choice for patients preferring tablets, once sufficient, robust data can inform the risk–benefit balance between low-molecular-weight heparin and direct oral anticoagulants.

## Appendix 1

### Topic guide

1. You’ve had cancer and also a blood clot, how has it affected your life/lives? (To patient and also partner/carer)
2. How was it when you had the blood clot and started on the trial?
3. How did you feel when you were first told you’d get an injection/pill?
4. Did you have a preference for the type of medication you received?
5. How were you introduced to the medication you might be given on the trial?
6. Do you have any previous experience of anticoagulants?
7. Can you tell me what you know about the 2 different drugs?
  – Which is the currently recommended drug?
  – Effectiveness of each drug.
  – Risks associated with each drug.
8. How are you finding using the medication you’ve been given for the clot? (To patient and also partner/carer)
9. How does it affect your daily lives? (To patient and also partner/carer)
10. How did it feel the first time you injected yourself?
  – Bruising, etc.
  – Did it bother you?
11. Have you experienced any bleeding since using the medication?
12. How does it feel knowing you may have a bleed due to the medication?
13. Have you had any more clots, how was that?
14. What’s it been like knowing you could get a blood clot again?
15. Overall what do you think of the drug you are taking?
  – Help or hindrance?

## References

[1] Blom JW Doggen CJ Osanto S et al Malignancies, prothrombotic mutations, and the risk of venous thrombosis. JAMA 2005; 293(6): 715–722. 15701913 10.1001/jama.293.6.715

[2] Noble S. The challenges of managing cancer related venous thromboembolism in the palliative care setting. Postgrad Med J 2007; 83(985): 671–674. 17989265 10.1136/pgmj.2007.061622 PMC2659959

[3] Prandoni P Lensing AW Piccioli A et al Recurrent venous thromboembolism and bleeding complications during anticoagulant treatment in patients with cancer and venous thrombosis. Blood 2002; 100(10): 3484–3488. 12393647 10.1182/blood-2002-01-0108

[4] Johnson MJ Sheard L Maraveyas A et al Diagnosis and management of people with venous thromboembolism and advanced cancer: how do doctors decide? A qualitative study. BMC Med Inform Decis Mak 2012; 12: 75. 22818215 10.1186/1472-6947-12-75 PMC3445826

[5] Sheard L Prout H Dowding D et al Barriers to the diagnosis and treatment of venous thromboembolism in advanced cancer patients: a qualitative study. Palliat Med 2013; 27(4): 339–348. 23093572 10.1177/0269216312461678

[6] Johnson M. How do palliative physicians manage venous thromboembolism? Palliat Med 1997; 11(6): 462–468. 9519169 10.1177/026921639701100606

[7] NCCN. Cancer-associated venous thromboembolic disease, https://www.nccn.org/professionals/physician_gls/f_guidelines.asp

[8] Farge D Bounameaux H Brenner B et al International clinical practice guidelines including guidance for direct oral anticoagulants in the treatment and prophylaxis of venous thromboembolism in patients with cancer. Lancet Oncol 2016; 17(10): e452–e466. 27733271 10.1016/S1470-2045(16)30369-2

[9] Lyman GH Bohlke K Khorana AA et al Venous thromboembolism prophylaxis and treatment in patients with cancer: American Society of Clinical Oncology clinical practice guideline update 2014. J Clin Oncol 2015; 33(6): 654–656. 25605844 10.1200/JCO.2014.59.7351 PMC4881372

[10] Deitcher SR Kessler CM Merli G et al Secondary prevention of venous thromboembolic events in patients with active cancer: enoxaparin alone versus initial enoxaparin followed by warfarin for a 180-day period. Clin Appl Thromb Hemost 2006; 12(4): 389–396. 17000884 10.1177/1076029606293692

[11] Hull RD Pineo GF Brant RF et al Long-term low-molecular-weight heparin versus usual care in proximal-vein thrombosis patients with cancer. Am J Med 2006; 119(12): 1062–1072. 17145251 10.1016/j.amjmed.2006.02.022

[12] Levine MN Lee AY Kakkar AK. Low-molecular-weight heparin versus oral anticoagulant therapy for the long-term treatment of symptomatic venous thromboembolism: is there any difference in cancer-related mortality? J Clin Oncol 2005; 23(28): 7248–7250. 10.1200/JCO.2005.01.7897 16192623

[13] Noble SI Shelley MD Coles B et al Management of venous thromboembolism in patients with advanced cancer: a systematic review and meta-analysis. Lancet Oncol 2008; 9(6): 577–584. 18510989 10.1016/S1470-2045(08)70149-9

[14] Watson HG Keeling DM Laffan M et al Guideline on aspects of cancer-related venous thrombosis. Br J Haematol 2015; 170(5): 640–648. 26114207 10.1111/bjh.13556

[15] Benelhaj NB Hutchinson A Maraveyas AM et al Cancer patients’ experiences of living with venous thromboembolism: a systematic review and qualitative thematic synthesis. Palliat Med 2018; 32(5): 1010–1020. 29485330 10.1177/0269216318757133

[16] Noble SI Finlay IG. Is long-term low-molecular-weight heparin acceptable to palliative care patients in the treatment of cancer related venous thromboembolism? A qualitative study. Palliat Med 2005; 19(3): 197–201. 15920933 10.1191/0269216305pm1008oa

[17] Mockler A O’Brien B Emed J et al The experience of patients with cancer who develop venous thromboembolism: an exploratory study. Oncol Nurs Forum 2012; 39(3): E233–E240. 22543394 10.1188/12.ONF.E233-E240

[18] Seaman S Nelson A Noble S. Cancer-associated thrombosis, low-molecular-weight heparin, and the patient experience: a qualitative study. Patient Prefer Adherence 2014; 8: 453–461. 24748774 10.2147/PPA.S58595 PMC3986276

[19] Noble S Prout H Nelson A. Patients’ Experiences of LIving with CANcer-associated thrombosis: the PELICAN study. Patient Prefer Adherence 2015; 9: 337–345. 25750522 10.2147/PPA.S79373 PMC4348145

[20] Noble SI Nelson A Fitzmaurice D et al A feasibility study to inform the design of a randomised controlled trial to identify the most clinically effective and cost-effective length of Anticoagulation with Low-molecular-weight heparin In the treatment of Cancer-Associated Thrombosis (ALICAT). Health Technol Assess 2015; 19(83): vii–xxiii. 10.3310/hta19830 PMC4781092 26490434

[21] Noble S Matzdorff A Maraveyas A et al Assessing patients’ anticoagulation preferences for the treatment of cancer-associated thrombosis using conjoint methodology. Haematologica 2015; 100(11): 1486–1492. 26294737 10.3324/haematol.2015.127126 PMC4825304

[22] Ritchie J Lewis J Nicholls CM et al Qualitative research practice: a guide for social science students and researchers. London; Thousand Oaks; New Delhi: SAGE, 2013.

[23] Hammersley M Atkinson P. Ethnography: principles in practice. 2nd ed. London: Routledge, 1995.

[24] Gale NK Heath G Cameron E et al Using the framework method for the analysis of qualitative data in multi-disciplinary health research. BMC Med Res Methodol 2013; 13: 117. 24047204 10.1186/1471-2288-13-117 PMC3848812

[25] Tong A Sainsbury P Craig J. Consolidated criteria for reporting qualitative research (COREQ): a 32-item checklist for interviews and focus groups. Int J Qual Health Care 2007; 19(6): 349–357. 17872937 10.1093/intqhc/mzm042

[26] APPTG. Venous thromboembolism (VENOUS THROM-BOEMBOLISM) in cancer patients Cancer, chemotherapy and clots, http://apptg.org.uk/wp-content/uploads/2016/12/VTE-in-Cancer-Patients-2016.pdf

[27] Kirwan C Nath E Byrne G et al Prophylaxis for venous thromboembolism during treatment for cancer: questionnaire survey. BMJ 2003; 327(7415): 597–598. 12969928 10.1136/bmj.327.7415.597 PMC194089

[28] Matzdorff A David G. Management of venous thromboembolism in cancer patients. Rev Vasc Med 2014; 2: 24–36.

[29] EWP. Cancer-associated thrombosis (CAT), a neglected cause of cancer death: actions needed to increase health outcomes and reduce mortality, https://www.cathrombosis.com/content/type/resources

[30] Bennett P Patterson K Noble S. Predicting post-traumatic stress and health anxiety following a venous thrombotic embolism. J Health Psychol 2016; 21(5): 863–871. 25030797 10.1177/1359105314540965

[31] Hunter R Lewis S Noble S et al ‘Post-thrombotic panic syndrome’: a thematic analysis of the experience of venous thromboembolism. Br J Health Psychol 2017; 22(1): 8–25. 27611117 10.1111/bjhp.12213

[32] Noble S Lewis R Whithers J et al Long-term psychological consequences of symptomatic pulmonary embolism: a qualitative study. BMJ Open 2014; 4(4): e004561. 10.1136/bmjopen-2013-004561 PMC3987719 24694625

[33] Bury M. Chronic illness as biographical disruption. Sociol Health Illn 1982; 4(2): 167–182. 10260456 10.1111/1467-9566.ep11339939

[34] Williams S Medicine and the body. London; Thousand Oaks; New Delhi: SAGE, 2003.

[35] Corbin J Strauss A. Managing chronic illness at home: three lines of work. Qual Sociol 1985; 8(3): 224–247.

[36] Young A. Anticoagulation therapy in selected cancer patients at risk of recurrence of venous thromboembolism: results of the select-d pilot trial. Atlanta, GA: The American Society for Haematology, 2017.

[37] Raskob GE van Es N Verhamme P et al Edoxaban for the treatment of cancer-associated venous thromboembolism. N Engl J Med 2018; 378(7): 615–624. 29231094 10.1056/NEJMoa1711948

[38] Noble S. Are new anticoagulants a safe and reasonable alternative to low molecular heparins? Thromb Res 2018; 164(Suppl. 1): S157–S161. 29703476 10.1016/j.thromres.2018.01.033

[39] Ward K Gott M Hoare K. Participants’ views of telephone interviews within a grounded theory study. J Adv Nurs 2015; 71(12): 2775–2785. 26256835 10.1111/jan.12748

[40] Seymour J Dix G Eardley T. Joint accounts: methodology and practice in research interviews with couples. York: Social Policy Research Unit, University of York, 1995.

[41] Arksey H Knight PT Interviewing for social scientists: an introductory resource with examples. London; Thousand Oaks; New Delhi: SAGE, 1999.

